# Diagnostic value of heart rate‐based methods for assessing cardiac autonomic neuropathy in prediabetes and diabetes

**DOI:** 10.14814/phy2.70610

**Published:** 2025-10-08

**Authors:** D. P. S. C. Oliveira, C. A. Q. S. Souza, A. C. Facchin, B. C. Mariano, L. P. Silva, T. Peçanha

**Affiliations:** ^1^ Graduate Program in Rehabilitation Science and Physical‐Functional Performance, Faculty of Physical Therapy Federal University of Juiz de Fora Juiz de Fora Minas Gerais Brazil; ^2^ Faculty of Physiotherapy Federal University of Juiz de Fora Juiz de Fora Minas Gerais Brazil; ^3^ Graduate Program in Physical Education, Faculty of Physical Education and Sports Federal University of Juiz de Fora Juiz de Fora Minas Gerais Brazil; ^4^ Department of Sport and Exercise Sciences, Manchester Metropolitan University Institute of Sport, Faculty of Science & Engineering Manchester Metropolitan University Manchester UK

**Keywords:** cardiac autonomic dysfunction, heart rate recovery, heart rate variability, subclinical neuropathy

## Abstract

This study tested the ability of simple heart rate (HR)‐based measures—namely, resting HR variability (HRV), post‐exercise HRV, and HR recovery (HRR)—to identify cardiovascular autonomic neuropathy (CAN) in individuals with prediabetes and type 2 diabetes (T2D). Fifty subjects with T2D or prediabetes participated in the study. CAN was diagnosed using five cardiovascular reflex tests: HR response to Valsalva, deep breathing, and standing; and blood pressure response to sustained handgrip and standing. Resting HRV was evaluated using time‐ (SDNN, RMSSD) and frequency‐domain analysis (total power, low‐[LF] and high‐frequency power [HF]). Post‐exercise HRV and HRR were assessed over 2 minutes after the Incremental Shuttle Walking Test. HRR was calculated as the difference between peak HR and HR at 1 and 2 min post‐test (HRR_1min_, HRR_2min_). ROC curve analysis showed that the resting HF‐HRV index had the strongest diagnostic performance among resting indices (sensitivity = 67.9%, specificity = 86.4%, AUC = 0.755, *p* = 0.001), while HRR_2min_ showed the best performance among the post‐exercise indices (AUC = 0.672, *p* = 0.042). Overall, resting HRV indices consistently outperformed post‐exercise HRR and HRV indices in diagnostic performance. The results suggest that resting HRV has strong potential for detecting CAN in individuals with prediabetes and T2D, and could be incorporated into CAN screening protocols.

## 1 INTRODUCTION

Type 2 diabetes (T2D) is a chronic metabolic disorder associated with a poor lifestyle and characterized by persistent hyperglycemia caused by deficits in insulin function (American Diabetes Association, 2020). T2D develops insidiously over years, often progressing through a stage of prediabetes during which subclinical metabolic alterations and impairments in insulin action gradually worsen, remaining undiagnosed until significant progression occurs (de Castro et al., 2021).

A critical complication of diabetes is cardiovascular autonomic neuropathy (CAN), characterized by impaired autonomic regulation of cardiovascular functions. Evidence suggests that CAN develops early in the disease process, as individuals with prediabetes may already exhibit cardiac autonomic dysfunction (Eleftheriadou et al., 2021). In diabetes, CAN is associated with increased cardiovascular morbidity and mortality (Chowdhury et al., 2021). A meta‐analysis found that individuals with diabetes and CAN have a threefold higher risk of future cardiovascular events and mortality in comparison with those without CAN (Chowdhury et al., 2021) Despite its clinical significance, CAN remains underdiagnosed in individuals with prediabetes and T2D. Although early symptoms such as fatigue, dizziness, or exercise intolerance may raise suspicion in the context of diabetes, they are often considered nonspecific and can be attributed to other diabetes‐related complications or common comorbid conditions. This, combined with limited awareness and the underutilization of standardized tests, contributes to the diagnostic oversight of CAN in diabetes (Lin et al., 2017). The lack of simple, rapid, and widely accessible diagnostic tools with broad application further compounds this issue (Cardoso et al., 2023; Spallone et al., 2011; Zylla et al., 2019).

Cardiovascular reflex tests (CARTs), also known as Ewing tests, are the reference standard for CAN diagnosis (Ewing & Clarke, 1982). These tests evaluate heart rate (HR) and blood pressure (BP) responses to autonomic stressors (i.e., standing up, Valsalva maneuver, deep breathing, handgrip exercise) and offer high sensitivity and specificity (Ewing & Clarke, 1982; Pafili et al., 2015). However, CARTs are time‐consuming (requiring approximately 25–30 min, though realistically 40–45 min when including time to explain procedures and ensure test conditions and physiological parameters are stabilized) (Ewing & Clarke, 1982; Pafili et al., 2015), and demand trained personnel and multiple procedures that may limit broader application. Some CARTs may also be unsuitable for certain groups. For instance, sustained handgrip tests may be difficult for patients with hand arthritis, and the Valsalva maneuver may be contraindicated for individuals with severe retinopathy due to increased risk of hemorrhage (Lavezzo et al., 2012). These limitations highlight the need for more accessible methods to enable broader application in diagnosing CAN in prediabetes and T2D.

Assessment of resting heart rate variability (HRV) has emerged as a complementary, noninvasive, and rapid tool for evaluating cardiac autonomic function in T2D (Benichou et al., 2018; Coopmans et al., 2020). Studies show that individuals with T2D exhibit reduced resting HRV, an indicator of autonomic dysfunction that may manifest even in early disease stages such as prediabetes (Benichou et al., 2018; Coopmans et al., 2020). However, while resting HRV is widely used, it primarily reflects autonomic modulation in a relatively undisturbed state and may not fully capture the extent of cardiac autonomic dysfunction under a broader range of physiological conditions. Given these limitations, exercise‐based assessments have been gaining growing attention as an accessible method for evaluating autonomic responsiveness to stress. Namely, post‐exercise cardiac autonomic function assessment via analysis of heart rate recovery (HRR) and post‐exercise HRV have been employed as valid and reliable methods for assessing cardiac autonomic reactivation and cardiovascular risk in different populations, including T2D (Bhati et al., 2019; Peçanha et al., 2017; White & Raven, 2014). Slower HRR and reduced post‐exercise HRV immediately after exercise are markers of cardiac autonomic, primarily parasympathetic, dysfunction (Peçanha et al., 2017) and serve as independent predictors of adverse cardiovascular outcomes, regardless of clinical condition or other risk factors (Krantz & Manuck, 1984; Peçanha et al., 2017).

Interestingly, recent studies suggest that assessment of post‐exercise cardiac autonomic function may reveal autonomic dysfunction not detectable at rest (Bhati et al., 2019), potentially complementing or providing additional diagnostic value beyond resting HRV. However, studies assessing and comparing the diagnostic performance of rest and post‐exercise HRV and HRR for detecting CAN in prediabetes and T2D are scarce. A previous study reported superior diagnostic performance of post‐exercise HRV compared to resting HRV in T2D (Bhati et al., 2019); however, this study assessed post‐exercise HRV following a maximal exercise test, which may not be feasible or safe for high‐risk patients and may pose challenges in resource‐limited settings. Additionally, the study did not include individuals with prediabetes, limiting the generalizability of the findings across the broader spectrum of diabetes pathophysiology.

The present study aimed to address the limitations of previous research by evaluating the performance of resting HRV and post‐submaximal exercise HRV and HRR in detecting CAN among individuals with prediabetes or T2D. The focus was on assessing these heart rate (HR)‐based parameters using simple linear methods and easily replicable protocols, including a submaximal field‐based exercise, with the aim of facilitating future field applications. The hypothesis was that post‐exercise HRV and HRR measurements would demonstrate superior diagnostic capacity compared to resting HRV in detecting CAN among individuals with pre‐DM and T2DM.

## 2 MATERIALS AND METHODS

### 2.1 Study design and ethics statement

This observational cross‐sectional study was conducted at the Laboratory of Cardiorespiratory and Metabolic Evaluation at the Faculty of Physical Therapy, Federal University of Juiz de Fora, Brazil. The study protocol was reviewed and approved by the Institutional Research Ethics Committee (approval number 6.090.527/ CAAE: 67245023.5.0000.5147) and adhered to the principles of the Declaration of Helsinki. All participants provided written informed consent prior to their participation.

### 2.2 Participants

Eligible participants included individuals of both sexes aged 18 years or older, living with prediabetes (i.e., fasting plasma glucose between 100 and 125 mg/dL or glycated hemoglobin [HbA1c] between 5.7% and 6.4%) or type 2 diabetes (T2D; i.e., fasting plasma glucose ≥126 mg/dL or HbA1c ≥6.5%; or undergoing treatment for diabetes). The diagnoses were confirmed through laboratory tests performed within the last 3 months and provided by the participants. Exclusion criteria included clinical conditions contraindicating the tests (e.g., severe retinopathy, symptomatic heart failure, unstable coronary artery disease, complex ventricular arrhythmias, use of beta‐blockers, pacemakers, or implantable cardioverter defibrillators, and musculoskeletal limitations preventing walking).

Participants were recruited from the University community by word‐of‐mouth or via advertisement (leaflets or posts on social media) and from a cohort of individuals that participated in an ongoing trial (Silva et al., 2024).

### 2.3 Data collection and procedures

All procedures occurred during a single morning visit (7:00 am–12:00 pm). Participants were instructed via telephone to avoid caffeine for 12 h, alcohol, and strenuous physical activity for 24 h, and to have a light meal 1 h before the visit. They were also advised to have adequate sleep the night prior to the testing.

Participants who presented hypoglycemia or hyperglycemia during the study test visit (capillary glucose <80 mg/dL; >250 mg/dL with ketonuria; >300 mg/dL) were rescheduled up to 2 times. If the condition persisted, the participant was excluded from our investigation.

#### 2.3.1 Participant screening and baseline assessments

Upon arrival, participants were informed of the procedures and signed the consent form. Data collection started with an interview that assessed health status, lifestyle habits, and sociodemographic characteristics, followed by the assessment of physical activity levels using the short version of the International Physical Activity Questionnaire (IPAQ) (Matsudo et al., 2001). Anthropometric measurements, including height, weight, and body mass index (BMI), were then recorded. Subsequently, maximal voluntary contraction (MVC) in sustained handgrip was evaluated three times, with each attempt separated by a rest period of 1 min to minimize fatigue. The highest MVC value recorded during the attempts was considered the maximal force achieved.

#### 2.3.2 Assessment of cardiovascular autonomic neuropathy

CAN was assessed using five CARTs (Ewing & Clarke, 1982), as detailed below. During the tests, heart rate and RR intervals were continuously recorded using a Polar V800 monitor (Polar Electro, Kempele, Finland). Blood pressure (BP) was measured using the auscultatory method with a calibrated sphygmomanometer (Premium®, Brazil) and a stethoscope (Littmann®, 3M, United States).

##### Heart rate response to Valsalva maneuver

Participants performed the maneuver by blowing into a mouthpiece attached to a mercury manometer, maintaining an expiratory pressure of 40 mmHg for 15 s. RR intervals were recorded from the beginning until 15 s post‐maneuver. The Valsalva ratio was calculated as the longest RR interval following the maneuver divided by the shortest RR interval during expiratory effort. The reference values utilized for the Valsalva ratio were as follows: normal (>1.21), borderline (1.11–1.21), and abnormal (<1.10) (Ewing et al., 1985; Ewing & Clarke, 1982).

##### Heart rate variation during deep breathing

Participants performed six deep breaths (5‐s inhalation, 5‐s exhalation). RR interval variations (highest and lowest RR intervals) were recorded for each cycle and expressed as the mean difference between highest and lowest RR intervals across six cycles. Reference values were as follows: normal (>15 bpm), borderline (11–14 bpm), and abnormal (<10 bpm) (Ewing et al., 1985; Ewing & Clarke, 1982).

##### Blood pressure response to sustained handgrip

Resting BP was measured three times consecutively, followed by three measurements at 1‐min intervals during a 3‐min sustained handgrip at 30% MVC. BP was measured on the nondominant arm. The difference between the highest diastolic BP during handgrip and the mean diastolic BP at rest was calculated. Reference values were as follows: normal (>16 mmHg), borderline (11–15 mmHg), and abnormal (<10 mmHg) (Ewing et al., 1985; Ewing & Clarke, 1982).

##### Immediate heart rate response to standing (30/15 ratio)

Participants rested in the supine position for 10 min. After that, they transitioned from supine to a standing position, and RR intervals were recorded from the moment they stood up until the 31st beat after standing. The 30/15 ratio was calculated as the RR interval at the 30th beat divided by the RR interval at the 15th beat after standing. The reference values for the 30/15 ratio were as follows: normal (>1.04), borderline (1.01–1.03), and abnormal (<1.00) (Ewing et al., 1985; Ewing & Clarke, 1982).

##### Blood pressure response to standing

This test was performed simultaneously with the previous test. BP was measured in the right arm. Supine BP was measured following 10 min of supine rest, while standing BP was measured 30–60 s after standing. The BP response to standing was calculated by subtracting supine systolic BP from the standing systolic BP (i.e., fall in systolic BP). Reference values were as follows: normal (<10 mmHg), borderline (11–29 mmHg), and abnormal (>30 mmHg) (Ewing et al., 1985; Ewing & Clarke, 1982).

##### CAN detection

CAN was detected based on the scores of the five CARTs above. For each test, scores were assigned as follows: normal (score = 0), borderline (score = 0.5), and abnormal (score = 1). Total scores ≥2 indicated the presence of CAN status (CAN+), while scores <2 indicated the absence of CAN (CAN−) (Ewing et al., 1985; Ewing & Clarke, 1982; Lin et al., 2017).

#### 2.3.3 Heart rate variability (HRV) at rest

Heart rate and RR intervals were continuously recorded using a V800 HR monitor connected to a chest strap (Polar®, Kempele, Finland). This device has been validated for measuring HRV both at rest and during physiological stress (Huang et al., 2021). Recordings began after 10 min of rest and lasted 10 min. Participants remained at rest in the supine position with the strap positioned on their chest. They were instructed to remain as relaxed as possible, not to talk, avoid moving or sleeping, and to keep their eyes open and breathe spontaneously for the test duration.

Resting HRV was analyzed using the Kubios HRV Standard software (v 2.0, Kuopio, Finland), in accordance with the guidelines of the Task Force (Malik, 1996). Each beat‐to‐beat RR interval was visually inspected using the software, and ectopic beats and/or artifacts were filtered using an automatic beat correction algorithm (medium threshold). A maximum error of 3% was accepted during the process. The signal was then scanned, and a continuous 5‐min segment with excellent signal stability and free of artifacts was selected for analysis. The following indices were calculated in the time domain: SDNN (standard deviation of the RR intervals in milliseconds); RMSSD (root mean square of the differences between successive RR intervals in milliseconds); pNN50 (percentage of successive RR intervals whose duration difference is greater than 50 ms); SD1 (standard deviation of instantaneous beat‐to‐beat RR interval variability, measured as the dispersion of points perpendicular to the identity line in a Poincaré plot, in milliseconds); SD2 (standard deviation of long‐term RR interval variability, measured as the dispersion of points along the identity line in a Poincaré plot, in milliseconds); SD1/SD2 (ratio between SD1 and SD2, reflecting the balance between short‐ and long‐term HRV). For analysis in the frequency domain, the data were resampled at 4 Hz and the trend component was removed using the smooth prior function (Tarvainen et al., 2002). The power spectral analysis was performed using the Fast Fourier Transform method (256 points, 50% overlap, Hanning window). The following indices were calculated in the frequency domain: VLF_abs_ (very‐low frequency spectral component in absolute units; <0.04 Hz); LF_abs_ (low‐frequency spectral component in absolute units; 0.04–0.15 Hz); HF_abs_ (high‐frequency spectral component in absolute units; 0.15–0.40 Hz); LF_nu_ (low‐frequency spectral component in normalized units); HF_nu_ (high‐frequency spectral component in normalized units); LF/HF (ratio between low‐ and high‐frequency spectral components); total power (sum of the power of all frequencies in the spectrum).

#### 2.3.4 Post‐exercise autonomic function analysis

Post‐exercise HRV and HRR were assessed following the Incremental Shuttle Walking Test (ISWT) (Singh et al., 1992), a submaximal field test commonly used to assess functional capacity in real‐world settings. Participants walked a 10‐m course marked by cones at each end, at increasing externally paced speeds regulated by audio beeps. The test continued until exhaustion (usually indicated by breathlessness or the inability to keep pace with the beeps and reach the cone in time) or until reaching 85% of their predicted maximum heart rate [210 − (0.65 × age)] (Singh et al., 1992). Upon completion of the test, participants were seated for a 2‐min recovery period during which recovery data were collected. HRR was calculated by subtracting the heart rate measured 1 min (HRR_1min_) and 2 min (HRR_2min_) (Peçanha et al., 2017) into recovery from the final heart rate reached at the end of the ISWT. Post‐exercise HRV indices were calculated using RMSSD, averaged over three time periods: the first minute (RMSSD_1min_), the second minute (RMSSD_2min_), and the entire 2‐min period (RMSSD_0–2min_) of recovery (Pradhapan et al., 2014). These indices were derived using the SinusCor software (Bartels et al., 2017).

#### 2.3.5 Order of the autonomic tests

The autonomic assessments were conducted in a fixed sequence to prioritize rest measurements before those involving physiological stress. HRV at rest was recorded first, followed by the CARTs, and then the ISWT and post‐exercise HRV and HRR assessments. The Polar V800 monitor strap was not removed at any point throughout the participant assessment process. However, to ensure accurate segmentation and signal specificity for each testing stage, a new recording was started on the Polar watch at the beginning of every test.

### 2.4 Statistical analysis

Data were analyzed using SPSS (v26.0). Continuous variables were expressed as mean ± SD or median (IQR), while categorical variables were expressed as frequency. Normality of the data distribution was tested using the Shapiro–Wilk test, while homogeneity of variances was assessed by the Levene test. Participants were grouped based on the presence or absence of CAN (i.e., CAN+ or CAN−). These groups were compared using the chi‐square test for categorical variables and *t*‐tests or Mann–Whitney *U* tests for continuous variables. The diagnostic performance of rest HRV and post‐exercise HRV and HRR was assessed by receiver operating characteristic (ROC) curve analysis. The area under the ROC curve was calculated and utilized to quantify the diagnostic performance of each measure. An AUC of 1.0 indicates perfect diagnostic accuracy, while an area equal to 0.5 reflects random diagnostic accuracy (Hajian‐Tilaki, 2013). Significance of the AUC was also assessed to determine whether the diagnostic performance of each measure was statistically significant. This analysis also provided calculations for sensitivity, specificity, true positives (TP), true negatives (TN), false positives (FP), and false negatives (FN) for various threshold values for each measure. The value with the highest Youden index (sensitivity + specificity − 1) was determined as the optimal cutoff point for each parameter evaluated. Based on the results from the ROC curve analysis, the positive predictive value (i.e., PPV = TP/(TP + FP)) and the negative predictive value (i.e., NPV = TN / (TN + FN)) were calculated. Significance was set at *p* < 0.05.

## 3 RESULTS

During the recruitment process, 108 individuals were identified through self‐referral and contacted by telephone or messaging app by the researchers or through targeted prescreening of a cohort of patients with T2D from a previous trial (Silva et al., 2024). These individuals were subsequently invited to attend the laboratory visit for testing. Among them, 27 individuals did not respond to contact attempts; 29 did not attend the scheduled study visit; and two were excluded because they had contraindications for testing. Therefore, 50 individuals (23 with prediabetes and 27 with T2D) were considered eligible and participated in the study.

CAN was present in 58% of the participants. Individuals with CAN (CAN+) were older and had a higher prevalence of hypertension and a previous COVID diagnosis in comparison with individuals without CAN (CAN−) (Table 1).

**TABLE 1:** Characterization of CAN+ and CAN‐ groups.

Variables	CAN+	CAN−	*p*
	(*N* = 29)	(*N* = 21)	
Age, years	61.28 ± 10.39	53.43 ± 11.22	0.014*
Male, *n* (%)	16 (55.2)	11 (52.4)	0.847
Insulin therapy, *n* (%)	3 (10.3)	2 (9.5)	0.925
Oral hypoglycemic, *n* (%)	23 (79.3)	19 (90.5)	0.293
Smoking, *n* (%)	0 (0)	0 (0)	1.00
HAS, *n* (%)	18 (62.1)	6 (28.6)	0.021*
Dyslipidemia, *n* (%)	15 (51.7)	8 (38.1)	0.424
CAV, *n* (%)	1 (3.4)	2 (9.5)	0.377
CAD, *n* (%)	4 (13.8)	3 (14.3)	0.961
AMI, *n* (%)	2 (6.9)	1 (4.8)	0.756
Peripheral neuropathy, *n* (%)	1 (3.4)	2 (9.5)	0.377
Diabetic retinopathy, *n* (%)	2 (6.9)	1 (4.8)	0.756
Musculoskeletal diseases, *n* (%)	14 (48.3)	10 (47.6)	0.964
COVID diagnosis, *n* (%)	19 (65.5)	5 (23.8)	0.004*
Weight (kg)	79.04 ± 18.03	78.09 ± 16.13	0.840
BMI (kg/m^2^)	28.76 ± 6.30	27.99 ± 3.87	0.623
SBP (mmHg)	125.17 ± 12.01	121.71 ± 13.70	0.348
DBP (mmHg)	76.21 ± 8.23	76.10 ± 13.63	0.971
Time since prediabetes or diabetes diagnosis (months)	89.62 ± 92.29	82.14 ± 61.39	0.748
Fasting blood glucose (mg/dL)	142.00 ± 64.78	133.89 ± 53.17	0.675
Capillary blood glucose (mg/dL)	153.38 ± 55.33	169.57 ± 84.77	0.418
HbA1c (%)	6.70 ± 1.08	7.08 ± 1.92	0.442
Total cholesterol (mg/dL)	183.65 ± 52.69	177.53 ± 44.99	0.718
LDL (mg/dL)	106.57 ± 50.14	112.92 ± 43.98	0.730
HDL (mg/dL)	47.83 ± 13.17	44.25 ± 10.91	0.398
Triglycerides (mg/dL)	136.47 ± 72.24	147.13 ± 76.80	0.686

*Note*: Continuous variables were expressed as mean ± SD. Categorical variables were expressed as frequency.

Abbreviations: AMI, acute myocardial infarction; BMI, body mass index; CAD, coronary artery disease; CVA, cerebrovascular accident; DBP, diastolic blood pressure; HAS, systemic arterial hypertension; HbA1c, glycated hemoglobin; SBP, systolic blood pressure.

* *p* ≤ 0.05: significant difference.

Table 2 presents the resting HRV and post‐exercise HRV and HRR data in CAN+ and CAN− individuals. For resting HRV, individuals with CAN+ presented reduced SDNN, RMSSD, SD1, HF_abs_, and HF_nu_, and increased LF_nu_ and LF/HF. As for the exercise testing, all participants were able to successfully perform the ISWT. In all cases, the test was terminated due to the inability to maintain the required pace, with no participant reaching 85% of their predicted maximum heart rate. The CAN+ group achieved a lower final heart rate, covered a shorter distance, and terminated the ISWT test earlier than the CAN− group. However, no significant differences were observed between the CAN+ and CAN− groups in any of the post‐exercise HRR or HRV indices (Table 2).

**TABLE 2:** Resting HRV, ISWT, Post‐exercise HRV and HRR.

Variables	CAN+	CAN−	*p*
	(*N* = 29)	(*N* = 21)	
Resting HRV			
SDNN (ms)†	19.90 [15.30–37.40]	30.20 [23.20–48.65]	0.038*
RMSSD (ms)†	15.00 [9.80–26.60]	24.60 [17.70–44.10]	0.016*
pNN50 (%)†	1.25 [0.00–4.64]	4.01 [0.65–22.49]	0.056
SD1 (ms)†	10.60 [7.00–18.80]	17.40 [12.50–31.25]	0.015*
SD2 (ms)†	22.50 [19.00–50.50]	40.00 [27.35–48.50]	0.069
SD1/SD2	0.51 ± 0.27	0.56 ± 0.24	0.452
VLF_abs_ (ms^2^)	87.68 ± 171.8	59.5 ± 53.09	0,420
LF_abs_ (ms^2^)†	143.00 [66.00–624.00]	377.00 [111.50–538.50]	0.201
HF_abs_ (ms^2^)†	73.00 [36.00–188.00]	267.00 [122.00–436.50]	0.002*
LF_nu_ †	76.89 [35.55–84.34]	54.00 [41.79–65.35]	0.038*
HF_nu_†	23.10 [15.65–64.44]	46.00 [34.64–58.20]	0.038*
LF/HF†	3.32 [0.55–5.38]	1.17 [0.71–1.88]	0.038*
Total Power (ms^2^)†	377.00 [72.00–1061.00]	682.00 [310.50–998.50]	0.093
Post‐exercise HRV and HRR			
ISWT			
Final HR (bpm)	108.71 ± 14.01	118.29 ± 16.23	0.032*
%Predicted HRmax (%)	63.48 ± 7.43	67.98 ± 10.22	0.081
Distance covered (m)	350.00 ± 88.39	409.05 ± 98.23	0.031*
Total test time (seconds)	373.28 ± 60.32	419.81 ± 60.75	0.010*
Post‐exercise data			
HRR_1min_	18.07 ± 7.38	18.85 ± 11.78	0.780
HRR_2min_	27.79 ± 9.84	32.30 ± 8.22	0.101
RMSSD_1min_†	7.28 [5.56–10.68]	7.78 [4.36–11.22]	0.917
RMSSD_2min_	9.45 ± 3.78	10.61 ± 6.08	0.424
RMSSD_0–2min_†	8.77 [6.12–12.33]	8.21 [5.29–12.83]	0.851

*Note*: j (*n* = 25). Continuous variables are presented as mean ± SD unless marked with a †, which indicates non‐normal distribution and data reported as median [interquartile range].

Abbreviations: abs, absolute; CAN−, cardiovascular autonomic neuropathy absent; CAN+, Cardiovascular autonomic neuropathy present; HF, high frequency power; HR, heart rate; HRmax, maximum heart rate; HRR, heart rate recovery delta; HRR, heart rate recovery; HRV, heart rate variability; LF/HF, high frequency flow and power ratio; LF, low frequency power; nu, normalized units; pNN50, percentage of consecutive pNN50 that differ by >50 ms; RMSSD, root mean square of successive differences between adjacent R‐R intervals; RMSSD_0–2MIN_, root mean square of the differences between successive heartbeats in ms the entire 2‐min period; RMSSD_1MIN_, root mean square of the differences between successive heartbeats in ms the first minute; RMSSD_2MIN_, root mean square of the differences between successive heartbeats in ms the second minute; SD1/SD2, ratio between the dispersion of points perpendicular to and along the identity line; SD1, scatter of points perpendicular to the identity line in ms; SD2, scatter of points along the identity line in ms; SDNN, standard deviation of RR interval; VLF, very low frequency power.

* *p* ≤ 0.05: significant difference.

**FIGURE 1:**
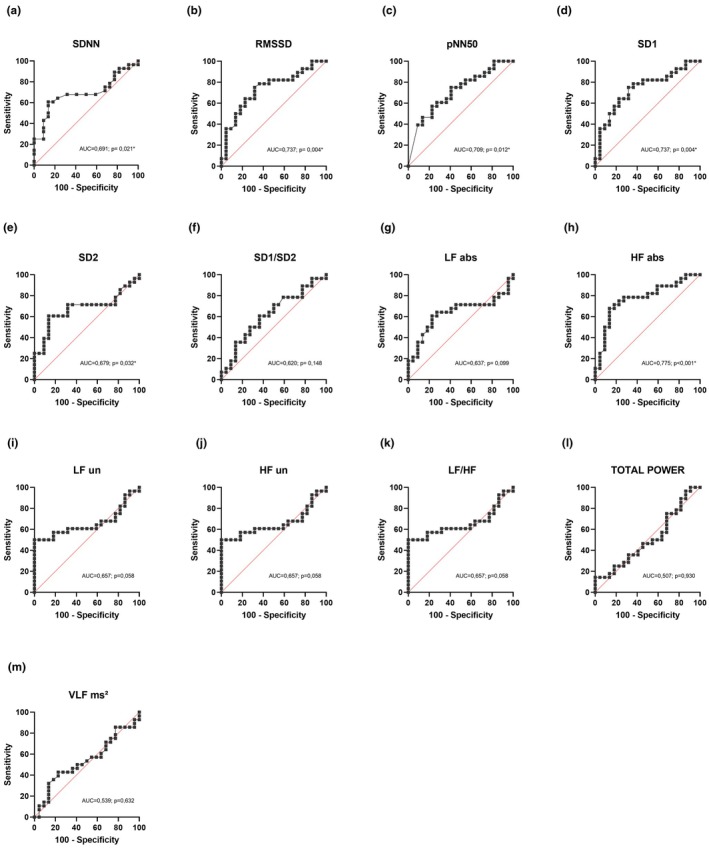
ROC curve analysis of the ability of resting HRV to detect CAN: *p ≤ 0.05: Significant difference; (a) SDNN, standard deviation of RR interval; (b) RMSSD, root mean square of successive differences between adjacent R‐R intervals; (c) pNN50, percentage of consecutive pNN50 that differ by >50 milliseconds; (d) SD1, scatter of points perpendicular to the identity line in ms; (e) SD2, scatter of points along the identity line in ms; (f) SD1/SD2, ratio between the dispersion of points perpendicular to and along the identity line; (g) low frequency HRV absolute units; (h) high frequency HRV absolute units; (i) low frequency HRV normalized units; (j) high frequency HRV absolute units; (k) LF/HF ratio; (l) total power HRV; (m) very low frequency power.

**FIGURE 2:**
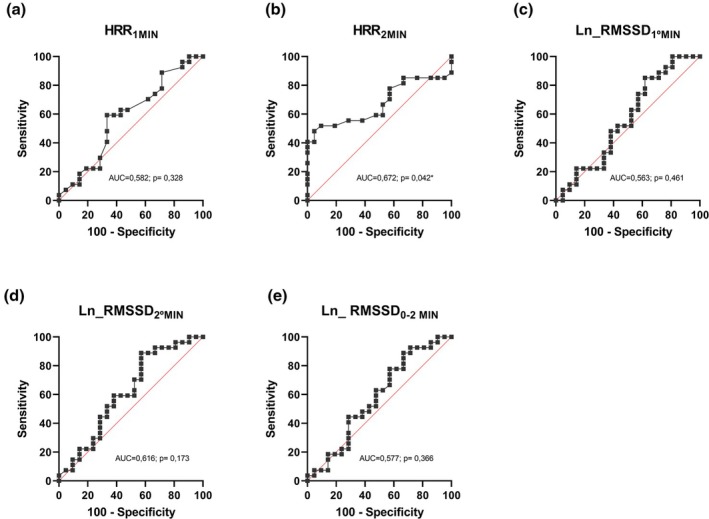
ROC curve analysis post‐exercise HRV and HRR to detect CAN: *p ≤ 0.05: Significant difference; (a) HRR_1min_, heart rate recovery 1 first minute; (b) HRR_2min_, heart rate recovery 2 second minute; (c) LnRMSSD_1MIN_, root mean square of the differences between successive heartbeats in ms the first minute; (d) LnRMSSD_2MIN_, root mean square of the differences between successive heartbeats in ms the second minute; (e) LnRMSSD_0–2MIN_, root mean square of the differences between successive heartbeats in ms the entire 2‐min period.

Figures 1 and 2 and Table 3 present the parameters of the ROC curve analysis, highlighting the diagnostic performance of each resting HRV, post‐exercise HRV, and HRR parameters in detecting CAN among the study participants. For resting HRV, the HF_abs_ index showed the best overall diagnostic performance (AUC = 0.775, *p* < 0.001; sensitivity = 67.9%, specificity = 86.4%, Youden Index = 0.543 for a cutoff of 114.00 ms^2^; Figure 1, Table 3). Other resting HRV indices also showed good diagnostic performance, either presenting high sensitivity (e.g., RMSSD and SD1) or high specificity (e.g., LF_nu_, HF_nu_, LF/HF, and total power) (Figure 1, Table 3).

**TABLE 3:** Diagnostic performance parameters for resting and post‐exercise HRV indices.

Variables	Sensitivity (%)	Specificity (%)	Youden	Cuttoff (≤)	PPV (%)	NPV (%)
Resting HRV						
SDNN (ms)	60.7	86.4	0.471	24.80	80.0	71.0
RMSSD (ms)	75.0	68.2	0.432	22.35	68.0	75.0
pNN50 (%)	57.1	77.3	0.344	1.31	70.0	66.0
VLF_abs_	14.2	95.4	0.100	126.0	80.0	46.7
LF_abs_	60.7	77.3	0.380	215.50	71.0	68.0
HF_abs_	67.9	86.4	0.543	114.00	82.0	75.0
LF_nu_	50.0	100.0	0.500	76.45^α^	100.0	69.0
HF_nu_	50.0	100.0	0.500	23.54	100.0	69.0
LF/HF	50.0	100.0	0.500	3.24^α^	100.0	69.0
Total power	14.3	100.0	0.143	49.50	100.0	56.0
SD1	75.0	68.2	0.432	15.80	68.0	75.0
SD2	60.7	86.4	0.471	32.20	80.0	71.0
SD1/SD2	60.7	63.6	0.243	0.44	60.0	64.0
Post‐exercise						
HRR_1MIN_	77.8	28.6	0.064	22.50	50.0	59.0
HRR_2MIN_	48.1	95.2	0.433	24.50	90.0	67.0
RMSSD_1min_	85.2	38.1	0.233	10.96	56.0	74.0
RMSSD_2min_	88.9	42.9	0.318	13.06	59.0	81.0
RMSSD_0–2min_	88.9	33.3	0.222	13.33	55.0	77.0

Abbreviations: abs, absolute; HF, high frequency power; HRR, heart rate recovery; HRV, heart rate variability; LF/HF, high frequency flow to power ratio; LF, low frequency power; NPV, negative predictive value; pNN50, percentage of consecutive pNN50 that differ by >50 ms; PPV, positive predictive value; RMSSD, root mean square of successive differences between adjacent R‐R intervals; RMSSD_0–2MIN_, root mean square of the differences between successive heartbeats in ms the entire 2‐min period; RMSSD_1MIN_, root mean square of the differences between successive heartbeats in ms the first minute; RMSSD_2MIN_, root mean square of the differences between successive heartbeats in ms the second minute; SD1/SD2, ratio between the dispersion of points perpendicular to and along the identity line; SD1, scatter of points perpendicular to the identity line in ms; SD2, scatter of points along the identity line in ms; SDNN, standard deviation of RR interval; un, normalized units; VLF, very low frequency power.

^α^ For the LF_nu_ and LF/HF indices consider cutoff point as ≥.

Diagnostic performance was consistently poorer for the post‐exercise indices (Figure 2, Table 3). Among them, the index that presented the best overall diagnostic performance was the HRR_2min_ (AUC = 0.672, *p* = 0.042; sensitivity = 48.1%, specificity = 95.2%, Youden Index = 0.433 for a cutoff of ≤24.5 bpm).

## 4 DISCUSSION

This study investigated the ability of simple, accessible HR‐based methods for detecting CAN in individuals with prediabetes and T2D. These methods included resting HRV, post‐submaximal exercise HRR, and post‐exercise HRV. Notably, this is the first study to evaluate the diagnostic performance of post‐exercise HRR and HRV for CAN detection following a submaximal exercise protocol, rather than a maximal exercise, offering a more feasible and scalable approach for real‐world settings. The inclusion of individuals with prediabetes also broadens the scope of CAN screening to earlier stages of metabolic dysfunction. Overall, the index with the greatest ability to detect CAN was the resting HRV‐HF_abs_.

The present study used a “staged approach” to test the ability of different HRV and HRR‐based indices to detect CAN in individuals with prediabetes and T2D. Firstly, these indices were compared between individuals presenting with CAN and those not presenting with CAN. Interestingly, while several resting HRV indices differed between CAN+ and CAN− individuals, no differences were observed for the calculated post‐exercise indices. Further testing using ROC analysis confirmed that the resting HRV indices outperformed the post‐exercise indices in terms of overall diagnostic value, with higher sensitivity, specificity, and AUC.

Data provided by the present study may help to identify indices with good ability for detecting CAN, which could be used in clinical practice. However, defining the “best” diagnostic index (or indices) is not a straightforward process and may depend on resources, context, and the purpose of the investigation. In general, an index with high sensitivity (the ability to correctly identify individuals with CAN) and specificity (the ability to correctly identify individuals without CAN) should likely be prioritized. In the present study, the resting HRV‐HF_abs_ index demonstrated the best combination of sensitivity and specificity. Values of resting HF_abs_ lower than 114 ms^2^ correctly identified 67.9% of the individuals with CAN, while values of HF_abs_ higher than 114 ms^2^ correctly identified 86.4% of individuals without CAN. The superior diagnostic value of the HF_abs_ index over other HRV indices supports the notion that spectral analysis of HRV, with subsequent separation into its main frequency components (VLF, LF, and HF), each providing distinct information about cardiac autonomic function, offers a more precise method for extracting physiologically specific data compared to time‐domain analysis, which is often considered less “physiologically specific” (Litvack et al., 1995; Malik, 1996). Relevant to the present study's findings, the HF index specifically reflects parasympathetic modulation coupled with respiratory activity. Alterations in this mechanism have been extensively described as a key component of CAN in DM (Freeman et al., 1995; Mackay, 1983; Smith, 1982), helping to explain why the HF index may be a good marker for identifying CAN in this population.

It is also important to consider the trade‐off between sensitivity and specificity when defining the best diagnostic index, meaning that one may need to be prioritized over the other depending on the clinical goal. For instance, indices with high specificity may be preferred when the primary goal is to ensure that individuals without CAN are not incorrectly diagnosed, thereby avoiding unnecessary treatments or follow‐up testing. In this case, resting HRV indices such as LF_nu_, HF_nu_, and LF/HF may be preferred, as they achieved 100% specificity (albeit with only 50% sensitivity). On the other hand, indices with higher sensitivity may be favored when the goal is identifying individuals who are likely to have CAN and should be referred for further testing. Interestingly, the post‐exercise HRV indices RMSSD_2min_ and RMSSD_0–2min_ outperformed all other indices (rest and post‐exercise) in terms of sensitivity. An RMSSD_2min_ equal to or lower than 13.06 ms and RMSSD_0–2min_ equal to or lower than 13.33 ms correctly identified 88.9% of individuals with CAN, meaning they can be effectively used for screening individuals who are likely to have CAN and should be referred for further diagnostic testing. These findings indicate that even though overall resting HRV data performs better than post‐exercise HRV and HRV to detect CAN, that is not necessarily true in all cases and for all applications. However, the reduced specificity (~40%) and PPV (<60%) of the above post‐exercise indices indicate that some individuals deemed as positive for CAN based on their provided cutoff points are likely false positives, meaning that a significant proportion of individuals who do not have CAN may be incorrectly identified as having the condition, potentially leading to unnecessary referrals.

The worse overall diagnostic performance of the post‐exercise HRV and HRR indices compared to resting HRV indices contrasts with the study hypothesis. In fact, a substantial body of medical literature supports the notion that cardiovascular responses to acute stressors, such as exercise, may be superior to resting assessments in detecting underlying cardiovascular abnormalities (Ireland et al., 2021; Koshy et al., 2019; Sveric et al., 2018). This concept is also reflected in the autonomic literature as recent studies have suggested that post‐exercise HRV measurements can reveal cardiac autonomic dysfunction that is otherwise undetected by resting measurements (Bhati et al., 2019; Lin et al., 2017). Using measures similar to those in the present study, Bhati et al. (2019) reported superior diagnostic performance of post‐exercise HRV over resting HRV to detect CAN in individuals with T2DM, directly contrasting with our findings. The characteristics of the exercise used to evaluate post‐exercise autonomic function may help explain the difference between the results of these previous studies and the present study. While most studies utilize maximal exercise (Bhati et al., 2019; Lin et al., 2017), the present study analyzed post‐exercise indices following an incremental externally paced, symptom‐limited submaximal test. We opted for a submaximal exercise protocol as it is more practical, safe, and easily applied in wider clinical and field settings, particularly in larger populations. However, interpretation of data coming from previous and the present study suggests that submaximal tests may be less sensitive to detect CAN than maximal tests. Further to that, it is important to underscore that in the present study individuals with CAN+ terminated the ISWT earlier and covered a lower distance compared to the CAN− group, reaching a lower heart rate at the end of the test. It is not possible to exclude the possibility that a reduced level of stress and effort during the test (as demonstrated by a trend for reduced % predicted maximal HR) contributed to the CAN+ group not exhibiting a lower HRR and HRV after exercise in comparison with the CAN− group, which may have concealed the potential of these indices to detect CAN. Future investigations may use submaximal exercise protocols designed to elicit the same relative level of effort for each individual based on their maximal capacity, as this may improve the ability to detect CAN irrespective of individual fitness levels.

The present study tested the ability of resting HRV, post‐exercise HRV, and HRR to detect CAN in individuals with prediabetes and T2D. Assessment of these indices is easier and less time‐consuming than performing the CARTs. Importantly, assessments of resting HRV can be completed in as little as 5 min, while post‐exercise HRV and HRR can be obtained within approximately 12–15 min, including the submaximal exercise and recovery period, making them more practical and scalable for broader clinical use. Additionally, advances in HR recording technology have enabled these HR‐based measurements to be implemented in smartwatches (Massoomi & Handberg, 2019), further enhancing their potential for widespread screening of CAN in T2D. These technological advancements allow for the assessment of CAN in individuals with prediabetes or T2D to extend into outpatient clinical settings and over prolonged periods. Despite still being in its infancy, the incorporation of these measures of cardiac autonomic (dys)function could provide comparable benefits to diabetes management as those brought by other field measures, such as continuous blood glucose monitors (Mian et al., 2019; Olczuk & Priefer, 2018). Future studies should test the utility of combining these HR‐based methods with other disease control measurements in the clinical management and prevention of diabetes. Integration of these different field tools has the potential to significantly improve prediabetes and diabetes management and patient prognosis.

This study has some limitations that must be acknowledged. Firstly, the findings of this study are not generalizable to other protocols of exercise (e.g., maximal exercise) or to other populations, including individuals with other types of diabetes (e.g., type 1 diabetes or gestational diabetes). Furthermore, despite including both individuals with prediabetes and T2D, separate ROC analyses in each of these groups were not possible due to the small sample sizes of each group (23 with prediabetes and 27 with T2D), which limited statistical power. For this reason, it was also not possible to ascertain whether the diagnostic performance differed according to DM group. Finally, in the present study, individuals with CAN were older, had a higher prevalence of hypertension and of previous COVID infection, and had a lower cardiorespiratory fitness than the individuals without CAN. While these variables affect resting and post‐exercise HRV and HRR, they are likely to affect all CARTs and therefore the classification of CAN to a similar extent. As such, their influence does not necessarily confound the analysis of HRV and HRR as tools to diagnose CAN, but rather reflects shared physiological factors underlying the condition and its diagnostic criteria. In addition, we did not include a healthy control group, as our primary objective was to evaluate diagnostic markers of CAN within individuals with prediabetes and T2D; however, comparisons with healthy populations could be valuable in future studies to quantify the absolute magnitude of autonomic dysfunction.

Several practical and methodological limitations should be considered when interpreting the autonomic analyses in this study. Resting and post‐exercise HRV and HRR were recorded using a heart rate monitor and analyzed without accounting for other physiological parameters, such as respiratory movements. The use of ECG data could enable the detection of ectopic beats that might otherwise go unnoticed with standard heart rate monitoring. Equally important, the continuous recording of respiratory movements would allow assessing the coherence between spectral power in the HF band against respiratory activity, providing a more comprehensive HRV analysis (Malik, 1996). Although direct respiratory measurements were not available, we estimated respiratory frequency from the HF peak, which is known to closely reflect breathing rate under normal conditions (Denver et al., 2007; Song & Lehrer, 2003) and found no significant difference between groups (CAN+ = 14.0 ± 4.3 breaths.min^−1^, CAN− = 14.4 ± 4.4 breaths.min^−1^, *p* = 0.77), suggesting that differences in the measured HRV indices are unlikely to be driven by respiratory rate. As this was a posthoc exploratory analysis and not based on direct respiratory measurements, we chose not to include it in the main study results, but have mentioned it here to acknowledge this limitation. In an ideal scenario, a comprehensive assessment of HRV including ECG, continuous arterial pressure, and respiratory parameters (e.g., tidal volume and respiratory frequency) would provide a more detailed and precise evaluation of autonomic function. However, the tools used in this study prioritize simplicity and accessibility, enhancing their practicality for routine clinical use. The same rationale justifies using “traditional” linear HRV indices in this study, as more novel approaches (e.g., HRV frequency analysis using heart rate instead of RR intervals) (Kania et al., 2024) or nonlinear methods (e.g., approximate entropy, detrended fluctuation analysis, and symbolic analysis), albeit promising for improving the assessment of autonomic function, have less well‐defined physiological validation and involve complex processing, limiting their routine clinical applicability. Finally, a methodological limitation of the spectral HRV analysis is the use of relatively short signal segments (256 points, equivalent to 64 s). While this does not affect the LF and HF bands, which were the main focus of our analysis, it may have influenced the estimation of VLF, total power, and normalized spectral indices.

## 5 CONCLUSION

The present study evaluated the ability of resting HRV and post‐exercise HRV and HRR to identify CAN in individuals with prediabetes and T2D. The findings suggest that resting HRV, particularly the HF_abs_ index, demonstrates strong potential for detecting CAN in this population. Data coming from this study may contribute to enhancing the diagnosis of CAN in clinical practice for individuals with prediabetes and T2D. Furthermore, the integration of these HRV indices into consumer technology (e.g., smartwatches) could further expand their application, making CAN screening more accessible and practical in real‐world settings.

## AUTHOR CONTRIBUTIONS

D.P.S.C.O, L.P.S, and T.P. conceived and designed the research. D.P.S.C.O, C.A.Q.S.S, B.C.M and A.C.F performed experiments and analyzed data. D.P.S.C.O, L.P.S, and T.P. interpreted the results of experiments. D.P.S.C.O prepared Figures. D.P.S.C.O wrote the first draft of the manuscript. D.P.S.C.O, L.P.S, and T.P. edited the manuscript. All authors approved the final version of the manuscript.

## FUNDING INFORMATION

D.P.S.C.O received support from the Coordenação de Aperfeiçoamento de Pessoal de Nível Superior—Brasil (CAPES), Finance Code 001. T.P. was supported by a grant from the Conselho Nacional de Desenvolvimento Científico e Tecnológico (CNPq; 406196).

## CONFLICT OF INTEREST STATEMENT

No conflicts of interest, financial or otherwise, are declared by the authors.

## Data Availability

Data will be made available upon reasonable request.
